# *Prdm9* Incompatibility Controls Oligospermia and Delayed Fertility but No Selfish Transmission in Mouse Intersubspecific Hybrids

**DOI:** 10.1371/journal.pone.0095806

**Published:** 2014-04-22

**Authors:** Petr Flachs, Tanmoy Bhattacharyya, Ondřej Mihola, Jaroslav Piálek, Jiří Forejt, Zdenek Trachtulec

**Affiliations:** 1 Department of Mouse Molecular Genetics, Institute of Molecular Genetics of the Academy of Sciences of the Czech Republic, v.v.i., Prague, Czech Republic; 2 Research Facility Studenec, Institute of Vertebrate Biology, Academy of Sciences of the Czech Republic, Brno, Czech Republic; Texas A&M University, United States of America

## Abstract

PR-domain 9 (*Prdm9*) is the first hybrid sterility gene identified in mammals. The incompatibility between *Prdm9* from *Mus musculus domesticus* (Mmd; the B6 strain) and the *Hstx2* region of chromosome (Chr) X from *M. m. musculus* (Mmm; the PWD strain) participates in the complete meiotic arrest of mouse intersubspecific (PWD×B6)F1 hybrid males. Other studies suggest that also semisterile intersubspecific hybrids are relevant for mouse speciation, but the genes responsible remain unknown. To investigate the causes of this semisterility, we analyzed the role of *Prdm9* and Chr X in hybrids resulting from the crosses of PWK, another Mmm-derived inbred strain. We demonstrate that *Prdm9* and Chr X control the partial meiotic arrest and reduced sperm count in (PWK×B6)F1 males. Asynapsis of heterosubspecific chromosomes and semisterility were partially suppressed by removal of the B6 allele of *Prdm9*. Polymorphisms between PWK and PWD on Chr X but not in the *Prdm9* region were responsible for the modification of the outcome of *Prdm9* - Chr X F1 hybrid incompatibility. Furthermore, (PWK×B6)F1 hybrid males displayed delayed fertility dependent on the *Prdm9* incompatibility. While the *Drosophila* hybrid sterility gene *Overdrive* causes both delayed fertility and increased transmission of its own chromosome to the offspring, the segregation of Chr X and the *Prdm9* region from the mouse (PWK×B6)F1 males was normal. Our results indicate extended functional consequences of *Prdm9* - Chr X intersubspecific incompatibility on the fertility of hybrids and should influence the design of fertility analyses in hybrid zones and of laboratory crosses between Mmm and Mmd strains.

## Introduction

When two populations are separated in nature for a sufficient time period, they may fail to produce a fertile offspring when intercrossed again [1]. This condition is called hybrid sterility. Hybrid sterility is one way of reproductive isolation, which deepens the separation of populations by inhibiting the exchange of genetic information. This separation can finally lead to the formation of a new (sub)species. The sterile hybrids are most often of heterogametic sex [1], the male in the case of mammals and flies. Despite great biological interest, only a few animal hybrid sterility genes have been discovered [2], [3], [4], [5], allowing study of the mechanisms of speciation.

Our model of hybrid sterility is based on two closely related subspecies of the house mouse, *Mus musculus musculus* (Mmm) and *M*. *m*. *domesticus* (Mmd). These subspecies are effectively reproductively isolated in Europe forming a narrow hybrid zone [6]. The Mmm subspecies has been represented in our model by the wild-derived inbred strain PWD, while the classical strain C57BL/6J (henceforth B6) has been used as a representative of Mmd. Crosses between a PWD female (Mmm) and a B6 (Mmd) male yield (PWD×B6)F1 azoospermic males but fertile females (see [7] for a review). The ((PWD×B6) ×B6)BC1 backcross analysis has revealed two major and an undefined number of minor loci causing hybrid sterility. One of the two major loci – *Hstx2–* was mapped to chromosome (Chr) X [8], [9], [10]. The other locus, *Hst1* on proximal Chr 17, was also detected in a different cross based on its polymorphism between B6 and another Mmd strain, C3H, which produces sperm-carrying F1 males with PWD [11], [12]. *Hst1* was identified by physical mapping, expression profiling, allelic sequencing, and transgenic rescue as the *Prdm9* gene [4], [13], [14], [15], [16]. *Prdm9* (PR-domain containing 9), also called *Meisetz* (Meiotic SET domain with Zinc fingers) encodes meiosis-specific histone 3 methyltransferase; the knock-out mice exhibit meiotic arrest in both sexes [17]. PRDM9 specifies the sites of recombination [18], [19] and directs the meiotic double-strand breaks away from transcription start sites [20].

The Bateson-Dobzhansky-Muller incompatibility (DMI) model suggests that the decreased fitness of inter(sub)specific hybrids is caused by incorrect interaction of diverged alleles that are not adapted to each other due to separate evolution [21], [22], [23]. The fertility of (PWD×B6)F1 azoospermic males has been rescued by both removal and overexpression of *Prdm9^B6^* [24], suggesting a dominant-negative DMI. The presence of sperm in the males resulting from the reciprocal cross that uses B6 females, (B6×PWD)F1 [4], as well as the absence of sperm in the offspring of B6.PWD-Chr X subconsomic females and PWD males [8] has suggested that one of the partners of *Prdm9^B6^* in the DMI(s) causing sterility of (PWD×B6)F1 males is located on Chr X. Recently, the locus has been named *Hstx2* and mapped to a 4.7 Mb interval of Chr X [10]. The role of Chr X in mouse speciation has been manifested by the limited degree of introgression of subspecies-specific alleles from Chr X across the hybrid zone [25], [26].

Reduced fertility but not always complete sterility has been detected in the offspring of crosses between other Mmm- and Mmd- derived strains [27], [28], [29], [30], as well as in backcrosses [31], [32], [33], [34] and in the hybrid zone [35], [36]. Although some of this phenotypic difference may be caused by polymorphisms in *Prdm9* or *Hstx2*, other hybrid sterility genes may be involved.

Therefore, we asked whether the *Prdm9^B6^-Hstx2^Mmm^* DMI plays a role in the phenotypes reducing F1 hybrid fitness other than complete meiotic arrest. To answer this question, we replaced the PWD strain with PWK, derived from Mmm mice caught near the site of origin of PWD, to generate intersubspecific (PWK×B6)F1 males [37]. In contrast to (PWD×B6)F1 males, (PWK×B6)F1 males carry a low amount of sperm. Using a portfolio of genetically modified mice and intersubspecific (sub)consomics, as well as mouse crosses of PWK, PWD, and B6, we addressed the role of *Prdm9* and Chr X^PWK^ by measuring multiple quantitative phenotypes associated with various stages of hybrid spermatogenesis. We conclude that the *Prdm9^Mmd^-Hstx2^Mmm^* DMI affects meiosis also in (PWK×B6)F1 male hybrids, but it is modulated by polymorphisms between PWD and PWK located on Chr X but not in the *Prdm9* region. Because some aged (PWK×B6)F1 males carried functional sperm, we inquired whether mouse hybrid sterility is accompanied by distorted transmission of Chr X or Chr 17.

## Methods

### Ethics Statement

Mice were bred at the Specific Pathogen-Free Facility of the Institute of Molecular Genetics in Prague and at the non-barrier facility of the Institute of Vertebrate Biology in Studenec. The animal care obeyed the Czech Republic Act for Experimental Work with Animals (Decree No. 207/2004 Sb, and the Acts Nos. 246/92 Sb and 77/2004 Sb) fully compatible with the corresponding regulations and standards of European Union (Council Directive 86/609/EEC and Appendix A of the Council of Europe Convention ETS123). The protocol was approved by the Committee on the Ethics of Animal Experiments of the Institute of Molecular Genetics (Permit Number 137/2009).

### Mice and Genotyping

The PWK/Ph and PWD/Ph strains (Prague Wild K and D, respectively) were developed from two non-overlapping sets of wild Mmm mice caught near Prague [37]. The genome of PWK/PhJ has been sequenced [38]. The STUP strain was derived from Mmm trapped in Studenec, 150 km from Prague [39], but it is now extinct. The C57BL/6J classical strain is mostly of Mmd origin [40].

The *Prdm9^tm1Ymat^* knock-out was made in 129P2/OlaHsd embryonic stem cells by replacement of the first five coding exons with LacZ [17] and carriers backcrossed ten times to B6 background resulting in the B6-*Prdm9^KO^* strain; the differential segment of Chr 17 extends from the position 6.4 to 21.1 Mb (NCBI37 assembly) as confirmed by SNP analysis using the Mega Mouse Universal Genotyping Array (Mega MUGA) chip custom service (Geneseek-Neogen, USA).

The B6-*Prdm9^C3H^* (B6*B10.C3H-*Hst1^f^*) congenic carries C3H polymorphisms at *Prdm9* and the differential segment of Chr 17 is 3.3 to 6.4 Mb in length [24].

The B6-BAC5 and B6-BAC21 mice carrying C3H/HeJ transgenes (CHORI-34-45F17 and CHORI-34-289M8, respectively) on B6 background have been described [4], [24]. Briefly, B6-BAC5 carries two copies of the *Prdm9^C3H^* transgene and the BAC21 transgene overlaps BAC5 but harbors two copies of N-terminally truncated *Prdm9*. Unlike BAC21, BAC5 rescues fertility of (PWD×B6)F1 hybrid males. The Mega MUGA SNP analysis confirmed the B6/B6 background except for regions of Chr 13 different for B6-BAC21 and B6-BAC5 that may carry the integrated transgenes.

The C57BL/6J-Chr #^PWD/Ph^/ForeJ (abbreviated as B6.PWD-Chr #) chromosomal substitution (consomic) and subconsomic strains have been described [41].

The description of crosses and genotypes states the female parent first, e.g., (PWK×B6)F1 indicates the F1 offspring of a PWK female and a B6 male.

PCR primers and conditions used for genotyping have been published [24], [41]. Briefly, microsatellite markers for loci located at Chr17 (*D17Mit164* at the position 3 Mb, *D17M13* at 13 Mb, *D17Zt642* at 15 Mb, *D17Ch07* at 15.6 Mb, *D17Zt334* at 16 Mb, *D17M21* at 21 Mb, *D17Mit33* at 35 Mb, *D17Mit123* at 94 Mb) and Chr X (*DXMit166* at 49 Mb, *DXMit92* at 58 Mb, *DXMit194* at 67 Mb, *DXMit44* at 77 Mb), as well as three-primer assay for the *Prdm9* knock-out at 15.7 Mb were utilized.

### Phenotyping and Statistics

Testicular weight of paired testicles (TW) and body weight (BW) were measured in adult males of the indicated age. Sperm was extracted from the entire (SCe) and/or caput (SCc) epididymides. Slides with surface-spread nuclei (chromosomal spreads) were made from adult testicular cells utilizing isotonic [42] or hypotonic [43] treatment. Indirect immunofluorescence was performed with antibodies described in the Results and footnotes of Tables; the antibodies were obtained from the same providers and used in the same way as described earlier [9], [24]. Section methods have also been published [10]. The significance of BW, TW, rTW (TW/BW), and SC data was evaluated using Welsch’s t-test and/or Wilcoxon rank sum test, and cellular phenotypes also with χ^2^ test. ANOVA and Tukey honestly significant difference test as implemented in the JMP program (http://www.jmp.com/) was utilized for the post-hoc age-dependency analysis of fertility parameters of STUP, B6, and their hybrids. The ARRIVE (Animals in Research: Reporting *In Vivo* Experiments) guidelines checklist and details are included as Text S1.

## Results

### (PWK×B6)F1 Males are Semisterile

**Figure 1:**
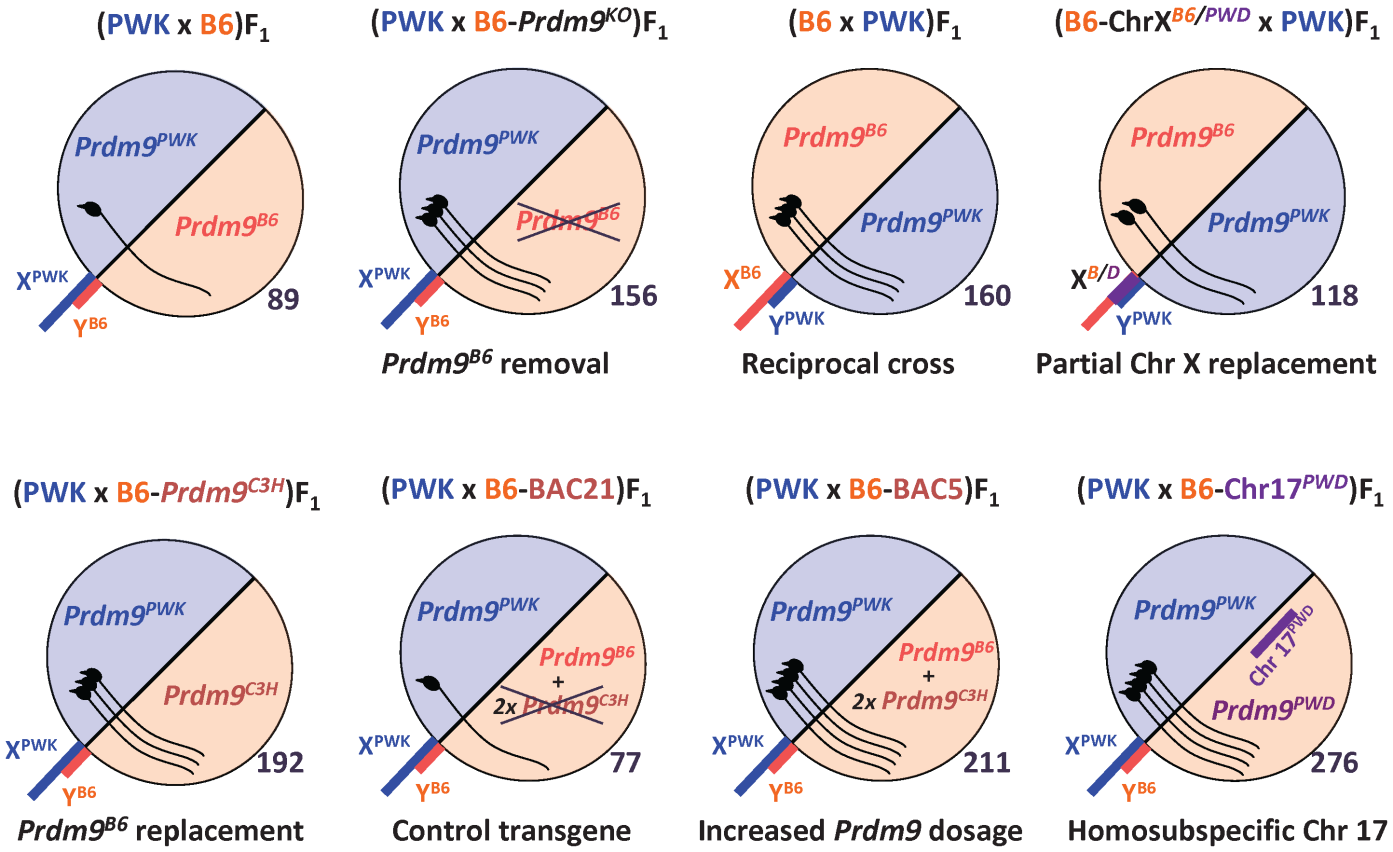
Schematic representation of fertility of F1 male offspring resulting from PWK crosses. The genotypes (female parents shown first) of each male are indicated. B6-*Prdm9^KO^*, congenic harboring *Prdm9* knock-out on B6 background; B6-ChrX*^B6/PWD^*, subconsomic B6.PWD-Chr X.3; B6-*Prdm9^C3H^*, B6 carrying C3H allele of *Prdm9*; B6-BAC21, B6 congenic carrying C3H transgene with truncated *Prdm9*; B6-BAC5, congenic carrying *Prdm9* transgene of C3H origin; B6-Chr17*^PWD^*, consomic B6.PWD-Chr 17. The circles symbolize genomes (sex chromosomes sticking out); the numbers on the lower right side of each circle represent the mean testicular weight (mg) at nine weeks of age; the pictures of spermatozoa within the circles symbolize the degree of fertility. See Tables 1 to 4 for exact values and other fertility parameters.

To assess the role of the incompatibility between *Prdm9* and ChrX from Mmm in the phenotypes reducing F1 hybrid fitness other than complete meiotic arrest, we focused on the (PWK×B6)F1 intersubspecific hybrids. Unlike (PWD×B6)F1 or (STUS×B6)F1 [37], [39], the (PWK×B6)F1 hybrid males carried sperm (Table 1). The (PWK×B6)F1 males had a lower relative testicular weight (TW) and sperm count in caput epididymis (SCc), and produced less pups than the reciprocal (B6×PWK)F1 (Figure 1, Table 1; p_rTW_<0.001, p_SCc_ = 0.036) or other fertile males or parental controls. The (PWK×B6)F1 hybrids yielded only 1.6 offspring per female per month (OFM) on average (Table 1). Moreover, their fertility was dependent on the age at which they could reproduce. Although nine of 13 (PWK×B6)F1 males tested produced pups, five that were mated starting at the age of nine weeks (when intrasubspecific mice are sexually mature) did not sire offspring until the average of 16 weeks of age. Thus the (PWK×B6)F1 males display oligospermia, decreased TW, and delayed fertility.

**Table 1:** The effect of *Prdm9* alleles and dosage on fertility parameters of PWK male hybrids.

Cross (female first)	*Prdm9*	Age	n	BW	TW	rTW	SCc	SCe	OFM
PWK×B6-*Prdm9^KO^*	PWK/B6	9	5	26	89	3.4	0.005	0.2	0 (n = 5)
PWK×B6-*Prdm9^KO^*	PWK/B6	17	4	29	104	3.2	0.08	3	1.6 (n = 6)
PWK×B6-*Prdm9^KO^*	PWK/−	9	3	22	156	7.0	1.8	10	5.3 (n = 3)
PWK×B6-*Prdm9^KO^*	PWK/−	20	2	27	188	5.9	2.2	27	8.0 (n = 4)
B6×PWK	B6/PWK	9	17	23	160	6.9	3.4	21	Na
B6×PWK	B6/PWK	20	8	25	160	6.5	3.8	na	4.9 (n = 3)
PWK×B6-*Prdm9^C3H^*	PWK/C3H	9	5	26	192	7.4	2.2	na	Na
PWK×B6-*Prdm9^C3H^*	PWK/C3H	14	4	28	202	7.3	2.4	na	4.8 (n = 1)
PWK×B6-BAC21	PWK/B6	9	3	25	77	3.2	0.002	na	Na
PWK×B6-BAC21	PWK/B6	17	1	22	103	4.6	0.5	na	Na
PWK×B6-BAC5	PWK/B6+2C3H	9	7	24	211	8.9	4.8	na	Na
PWK×B6-BAC5	PWK/B6+2C3H	17	6	24	206	8.5	4.2	na	Na
PWK×B6.PWD-Chr 17	PWK/PWD	9	3	30	276	9.3	5.0	na	Na
PWK×B6.PWD-Chr 17	PWK/PWD	16	3	34	303	9.0	8.1	na	Na

*Prdm9*, genotype at the *Prdm9* locus (maternal/paternal); −, null *Prdm9* allele; +, transgenic *Prdm9* allele(s); Age, male age (weeks); n, number of males; BW, average body weight (g); TW, mean weight of paired testicles in mg; rTW, relative TW (in mg per gram of BW); SC, average sperm count (millions) in paired caput epididymides (SCc) or in both entire epididymides (SCe); OFM, offspring per female per month; na, not analyzed; B6-*Prdm9^KO^*, heterozygote for *Prdm9* knock-out on B6 background; B6-*Prdm9^C3H^*, congenic carrying C3H allele of *Prdm9*; B6-BAC21, B6 congenic carrying C3H transgene with truncated *Prdm9*; B6-BAC5, B6 congenic carrying *Prdm9* transgene of C3H origin. See Figure 1 for a schematic view and the text of the Results section for statistical evaluations.

### Partial Age-dependent Meiotic Arrest in (PWK×B6)F1 Hybrid Males

**Figure 2:**
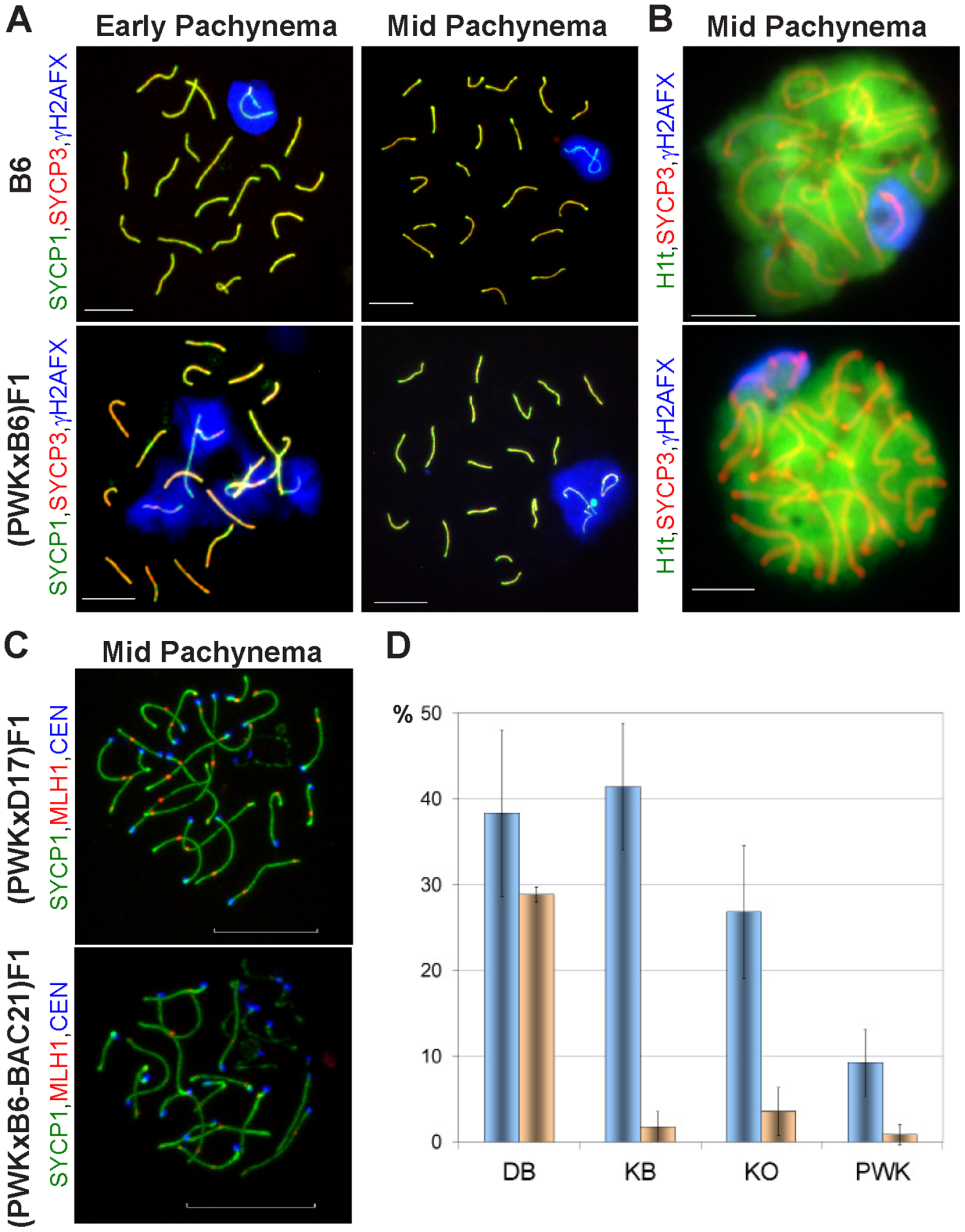
Phenotypes of primary spermatocytes from 9-week-old control and hybrid testes. The frequency of these phenotypes (Tables 2 and 3) determines the efficiency of meiosis. (**A**) Sex body formation and autosomal synapsis in pachynemas analyzed by SYCP1, SYCP3, and γH2AFX immunostaining on chromosomal spreads. Left, early pachynema; right, mid-pachynema; upper left and right, normal sex body and all chromosomes synapsed (abbreviated SB in Table 3); lower left, no sex body with multiple asynapsies (0B); lower right, abnormal sex body (AB, a sex body containing one or two unsynapsed autosomes). Bar, 10 µm. (**B**) Normal (upper) and abnormal (lower) primary spermatocytes labeled using antibodies against SYCP3 and histone variants H1t and γH2AFX (chromosomal spreads). Bar, 10 µm. (**C**) Meiotic recombination as a marker of meiotic progress; assessed by SYCP1, MLH1 recombination nodules and centromere (CEN) immunolabeling. Confocal microscopy of testicular nuclei prepared with isotonic buffer using semisterile (PWK×B6-BAC21)F1 and control (PWK×B6.PWD-Chr 17)F1 ((PWK×D17)F1) mice; upper, pachytene nucleus with all chromosomes synapsed and completed recombination (carrying over 20 MLH1 recombination nodules); lower, pachynema containing asynapsed chromosomes and containing less than 20 MLH1 nodules. Bar, 10 µm. (**D**) Autosomal asynapsis in PWK and PWD F1 males. Two classes of asynapses are plotted (mean ± standard deviation). Class I, blue columns, pachytene nuclei carrying one to three unsynapsed autosomes per nucleus (% of total pachynemas); class II, orange columns, % pachynemas with four to seven asynapses. The frequency of autosomal asynapsis was determined from the AB (A, lower right) and 0B (A, lower left) pachynemas. See Table 3 for other classes. DB, (PWD×B6)F1; KB, (PWK×B6)F1; K0, (PWK×B6)F1 with deleted *Prdm9^B6^* (*Prdm9^PWK/−^*); PWK, parental control. Class I asynapses decreased significantly in (PWK×B6)F1 upon *Prdm9^B6^* deletion (p = 0.037), but did not reach the level of PWK (p = 0.022). Class II asynapses were present in a significantly higher number in (PWD×B6)F1 than in all three other types of males, because all three probabilities (adjusted for multiple testing) were below 0.006 (Welsch’s t-test).

The (PWD×B6)F1 males suffer from a complete arrest of meiotic prophase I at the pachytene stage [9], [11]. To determine the reason for semisterility of the (PWK×B6)F1 males, indirect immunofluorescence of surface-spread nuclei (chromosomal spreads) from adult (PWK×B6)F1 testes was used to count the relative number of primary spermatocytes (Figure 2A,B, Table 2). This test can reveal the presence of late pachytene meiotic arrest, which causes an increased abundance of leptonemas, zygonemas, and early pachynemas at the expense of late pachynemas and diplonemas. The 9-week-old (PWK×B6)F1 males had the cellular composition intermediate between and significantly different from both the fertile control (B6, p = 0.006) and from the (PWD×B6)F1 male (p<0.001) with completely arrested meiosis. To assess whether the delayed reproduction capability of the (PWK×B6)F1 males correlates with the strength of the meiotic arrest, we analyzed chromosomal spreads of 9- versus 20-week-old males using meiotic markers (Figure 2A, Table 3). These markers included synaptonemal complex proteins SYCP1 and SYCP3 to stage the cells and phosphorylated histone H2AX to detect the sex body, a round nuclear structure that encompasses the transcription-inactive sex chromosomes [44]. Meiotic arrest in (PWD×B6)F1 hybrids increases the frequency of pachynemas without sex body [4], [24] and of pachynemas with an abnormal sex body encompassing unsynapsed autosomes [9]. Meiotic progress determined as the percentage of normal pachynemas (containing normal sex body without autosomal and XY asynapses) improved significantly with the age of (PWK×B6)F1 hybrids (from 42 to 55%, p = 0.037, Table 3), suggesting that the delayed fertility was caused by a factor(s) regulating meiotic arrest.

**Table 2:** Cellular composition of primary spermatocytes from adult testes of F1 males.

Cross (female first)	Age	N	%Lep	%Zyg	%EP	%MP	%LPD
B6×B6	9	159	6	8	16	41	29
PWD×B6	9	200	18	21	43	16	2
PWK×B6	9	149	8	14	29	34	15
B6.PWD-Chr X.1×PWD	9	125	3	7	36	28	26
B6.PWD-Chr X.1×PWK	9	108	3	9	25	34	29
B6.PWD-Chr X.1s×PWD	9	138	16	28	39	16	1
B6.PWD-Chr X.1s×PWK	9	109	8	10	39	30	13
B6×B6	25	82	1	4	17	44	34
B6.PWD-Chr X.1s×PWD	25	90	7	26	41	23	3
B6.PWD-Chr X.1s×PWK	25	90	2	6	29	43	20

Age, male age (weeks); N, number of cells (a total from three males); Lep, Leptonema; Zyg, Zygonema; EP, Early pachynema; MP, Mid-Pachynema; LPD, Late Pachynema-Diplonema. Analyzed using antibodies against SYCP3, H1t, and γH2AX on chromosomal spreads (see Figure 2 for representative images). The control data on 9-week-old (B6×B6), (PWD×B6)F1, and (B6.PWD-Chr X.1(s) ×PWD)F1 have been published [10]. See Results for statistical evaluations.

**Table 3:** Pachytene phenotypes of PWK F1 males.

Cross	*Prdm9*	Age	N	SB	AB	0B
PWK×B6-*Prdm9^KO^*	PWK/B6	9	137	42	27	31
PWK×B6-*Prdm9^KO^*	PWK/B6	20	115	55	17	28
PWK×B6-*Prdm9^KO^*	PWK/−	9	107	69	15	16
PWK×B6-*Prdm9^KO^*	PWK/−	20	127	68	17	15
PWK×B6-BAC21	PWK/B6	14	54	39	28	33
PWK×B6.PWD-Chr 17	PWK/PWD	12	66	82	12	6
PWK×PWK	PWK/PWK	9	58	88	7	5
PWK×PWK	PWK/PWK	16	61	92	5	3

Age, male age (weeks); N, number of counted pachynemas from a total of two or three males; SB, AB, 0B, % pachytene spermatocytes carrying a normal sex body and all chromosomes synapsed (SB), an abnormal sex body (AB, a sex body containing unsynapsed autosomes), and neither abnormal nor sex body (0B; always carried also unsynapsed chromosomes). See Figure 2A for representative phenotypes and Table 1 for other abbreviations. Antibodies against SYCP3, SYCP1, and γH2AX were used to stage the cells on chromosomal spreads. See the text for statistical evaluations.

### The Semisterility of (PWK×B6)F1 Males is under the Control of Chr X and Chr 17

The epistatic interaction of the proximal Chr 17^PWD/B6^ with central Chr X^PWD^ is necessary for the sterility of (PWD×B6)F1 hybrids [8]. To determine the effect of these two regions on hybrid semisterility of the (PWK×B6)F1 males, fertility parameters were analyzed in 9-week-old reciprocal F1 and backcross males. Reciprocal (B6×PWK)F1 hybrids displayed better fertility parameters than the (PWK×B6)F1 males (Table 1), suggesting a role for Chr X^PWK^ in hybrid semisterility. However, alternative explanations exist, including the effects of Chr Y, mitochondrial genome, and differentially imprinted autosomal gene(s). To analyze the candidate loci responsible for fertility of (PWK×B6)F1 hybrids, we used backcross analyses. In case of two loci affecting fertility with an additive effect we would expect 25% of N2 males with an F1-like phenotype. All 20 phenotyped ((PWK×B6) ×PWK)N2 males were fertile and were not investigated in detail. Only three of 65 ((PWK×B6) ×B6)N2 males displayed the F1-like phenotype, suggesting the involvement of at least three genomic loci. Fertility co-segregated with Chr X and Chr 17, as animals carrying the combined genotype of PWK/B6 at proximal (but not distal) Chr 17 with PWK (n = 15) at a region of Chr X (49 to 77 Mb) had significantly lower rTW (mean 6.6 mg/g) and SCc (mean 2.2 millions) than the other N2 males (n = 31, 7.7 mg/g, p_rTW_ = 0.022; 3.5 million, p_SCc_ = 0.015). Thus the interaction of these two chromosomal regions might affect the fertility of both (PWK×B6)F1 and (PWD×B6)F1 males.

### Heterosubspecificity of Chr 17 is Involved in Semisterility of (PWK×B6)F1 Males

The replacement of Chr 17^B6^ with Chr 17^PWD^ rescues fertility of the sterile (PWD×B6)F1 hybrid [8] and of the semifertile (B6×PWD)F1 hybrid [24]. The effect of Chr 17^PWD^ on semisterility of the (PWK×B6)F1 hybrid was verified by comparing the phenotypes of the (PWK×B6)F1 males with the F1 male offspring of a PWK female and a B6.PWD-Chr 17 consomic male. The replacement of Chr 17^B6^ with Chr 17^PWD^ restored fertility of (PWK×B6)F1 in 9-week-old males (p_rTW_<0.001; p_SC_ = 0.017; Table 1, Figure 1). To determine the effect of B6/B6 homozygosity of Chr 17 on the fertility of PWK hybrids, the ((PWK×B6)×B6)N2 males carrying homozygous versus heterozygous proximal Chr 17 were compared. Fertility parameters of the N2 males harboring the B6/B6 genotype on proximal Chr 17 (n = 24; mean TW = 194 mg, SCc = 3.6 millions) were higher than those of the Chr 17^PWK/B6^ N2 males (n = 29; TW = 178 mg, p_TW_ = 0.030; SCc = 2.7 millions, p_SC_ = 0.045). Therefore, the heterosubspecificity of Chr 17 is probably important for the semisterility of (PWK×B6)F1 males.

### Fertility Rescue of (PWK×B6)F1 Hybrids by a change of Allele or Dosage of *Prdm9*

Proximal Chr 17 encompasses the *Prdm9* hybrid sterility gene [4]. To directly test the involvement of *Prdm9* in the semisterility of (PWK×B6)F1 hybrids, we analyzed the reproductive phenotypes of the F1 male offspring of PWK females and B6 male congenics carrying various *Prdm9* alleles and transgenes (Table 1, Figure 1). The fertility of the (PWK×B6)F1 males was rescued by a transgene carrying two copies of the C3H alleles of *Prdm9* and flanking genes (BAC5; p_rTW_<0.001; p_SCc_ = 0.001), but not by a transgene carrying two copies of the same genes except for truncated *Prdm9* (BAC21). An allelic replacement of the *Prdm9^B6^* allele using the B6-*Prdm9^C3H^* congenic strain also led to improved TW and SCc of (PWK×B6-*Prdm9^C3H^*)F1 animals (p_rTW_ = 0.003; p_SCc_ = 0.010). Removal of the *Prdm9^B6^* allele utilizing a *Prdm9* knock-out (B6-*Prdm9^KO^*) increased the fertility parameters of the (PWK×B6)F1 hybrids (p_rTW_<0.001; p_SCc_<0.001). The analyses of chromosomal spreads from adult testes indicated that the partial meiotic arrest was alleviated in the (PWK×B6)F1 males with removed *Prdm9^B6^* [(PWK×B6)F1-*Prdm9^PWK/−^*] compared to their *Prdm9^PWK/B6^* littermates (Table 3), as the relative number of pachynemas carrying a sex body and all chromosomes synapsed was higher (p<0.001); however, the meiosis in (PWK×B6)F1-*Prdm9^PWK/−^* was only partially rescued, as it failed to reach the level seen in the parental control (PWK, p<0.001). The same order applied to the extent of autosomal asynapses (Figure 2D). In contrast to (PWD×B6)F1 males, pachynemas containing more than four asynapsed autosomes per cell were rare in (PWK×B6)F1, (PWK×B6)F1-*Prdm9^PWK/−^*, and PWK males.

Another way to quantify the meiotic arrest in F1 hybrids is to analyze the ratio of pachytene spermatocytes with completed recombination to all pachynemas (Figure 2C); this ratio is reduced in some F1 hybrid testes due to meiotic arrest [9], [24]. Pachytene nuclei with completed recombination carry over 20 recombination nodules; these nodules can be detected as foci labeled by an antibody against the MutL homolog 1 (MLH1) protein. Meiotic progress determined in this way was near to normal in fertile (PWK×B6.PWD-Chr 17)F1 with Chr 17*^PWK/PWD^* (63% compared to 67% in B6), but remained partially arrested in semisterile (PWK×B6-BAC21)F1 carrying the BAC21 transgene with truncated *Prdm9* (25 of 40 versus 12 of 41, p = 0.003). The same conclusion was reached using another criterion of meiotic progress, the relative number of pachynemas carrying a normal sex body and all 19 autosomes synapsed (p<0.001, Table 3).

To explore whether the delayed onset of fertility can be relieved by manipulation of the dosage or allele of *Prdm9*, we analyzed the adult F1 males resulting from the crosses of PWK and various B6 congenics at two time points (Table 1). Delayed fertility was not detected in any of the four sets of (PWK×B6)F1 males with fertility rescued by removal, addition or replacement of *Prdm9* or Chr 17, as the TW and SC of these rescued hybrids were similar in young versus aged adults (Table 1). Moreover, the (PWK×B6)F1-*Prdm9^PWK/−^* males sired their first litter on average at 10 weeks of age (much earlier than the (PWK×B6)F1 hybrids at 16 weeks; p = 0.017) and their meiotic progress was similar at nine versus 20 weeks of age (p = 0.813, Table 3).

Thus, *Prdm9* is one of the causes of partial meiotic arrest and delayed fertility in (PWK×B6)F1 males.

### PWK versus PWD Differences on Chr X but not on Chr 17 Modulate Hybrid Sterility

We next investigated the genetic differences underlying the variation of fertility between (PWK×B6)F1 and (PWD×B6)F1 males using males resulting from the crosses ((PWK×PWD)×B6) and ((PWD×PWK)×B6) that were phenotyped at about 11 weeks of age and genotyped for two loci from Chr 17 and four loci from Chr X. Twenty of 75 and six of 13 animals from these crosses were azoospermic ((PWD×B6)F1-like), respectively. Although these crosses generated different proportions of azoospermic males (27% versus 46%), this difference was not statistically significant (p = 0.155) and both populations were combined for analysis. Markers for proximal Chr 17 loci polymorphic between PWK and PWD segregated randomly with the fertility phenotype in the tested offspring of these crosses, suggesting that the difference in sterility of (PWD×B6)F1 versus semisterility of (PWK×B6)F1 cannot be attributed to the Mmm Chr 17 or *Prdm9*. In other words, the alleles of hybrid sterility genes of PWD and PWK seem to be the same on Chr 17. Indeed, the *Prdm9* alleles of the PWD and PWK strains could not be distinguished by sequencing their coding regions [4], [19], [38].

Only six of 30 tested Chr X microsatellite markers mapping to four distinct loci (positions from 49 to 77 Mb) were polymorphic between PWK and PWD. The mapping of these loci revealed a significant correlation of TW and SCc with Chr X in the males resulting from the ((PWD×PWK)×B6) and ((PWK×PWD)×B6) crosses (p_TW_<0.001, Welsch’s t-test; p_SC_<0.001, Wilcoxon rank sum test; at position 67 Mb). This locus is thus close to or identical with *Hstx2* described previously [9], [10]. Because two of 42 animals carrying the PWD allele in this region displayed sperm and 16 of 39 males (41%) harboring the PWK allele were azoospermic, there is probably one or more additional loci polymorphic between PWD and PWK affecting fertility of their hybrids.

These data indicate that polymorphisms in the *Hstx2* region and additional loci, but not at proximal Chr 17, may explain the variance in hybrid sterility between (PWD×B6)F1 and (PWK×B6)F1.

### Multiple Chr X Loci Affecting Semisterility of (PWK×B6)F1 Hybrids

To refine the mapping of the X-controlled semisterility of (PWK×B6)F1 male hybrids, B6.PWD-Chr X# subconsomic strains [41] were used to exchange parts of Chr X in (B6.PWD-Chr X#×PWK)F1 males (Table 4). Only one subconsomic region (X.1), harboring B6 alleles from the position 62.1 Mb to the distal end of Chr X, rescued fertility to a degree similar to (B6×PWK)F1 males carrying Chr X*^B6^* (Table 4). In contrast, two (X.1 and X.3) of these three subconsomics crossed with PWD males rescued the resulting F1 males from azoospermia in experiments mapping the locus controlling meiotic arrest in (PWD×B6)F1 [8], restricting this locus to the region between positions 61.0 and 94.3 Mb. In other words, the X.2^PWD^ region ensures the (PWD×B6)F1-like sterility on both (PWK×B6)F1 and (PWD×B6)F1 backgrounds and the X.3^PWD^ region causes (PWK×B6)F1-like semisterility when combined with the (PWK×B6)F1 set of autosomes. Thus, at least two loci of Chr X might contribute to the semisterility of (PWK×B6)F1, one of them in the same region as *Hstx2*, [8], [9], [10] and the other distal to it. One or both of these loci may differ between PWK and PWD. These data also excluded the interactions of Chr Y, mitochondrial genome, and differentially imprinted autosomal genes as the causes of the asymmetric difference in fertility of the F1 males resulting from the reciprocal crosses of PWK and B6.

**Table 4:** Fertility phenotypes of 9-week-old PWK F1 hybrids.

Cross (female first)	TW	SCe	n
PWK×B6	110*	2*	12
B6×PWK	160	21	17
B6.PWD-Chr X.1×PWK	174	30	11
B6.PWD-Chr X.2×PWK	71**	0**	10
B6.PWD-Chr X.3×PWK	118*	9*	8

*p<0.05 and **p<0.001, compared to (B6.PWD-Chr X.1×PWK)F1; only the B6.PWD-Chr X.2 females produce completely sterile F1 with PWD males [8].

### Delayed Fertility in PWK Hybrids Carrying the *Hstx2^PWD^* Region of Chr X

**Figure 3:**
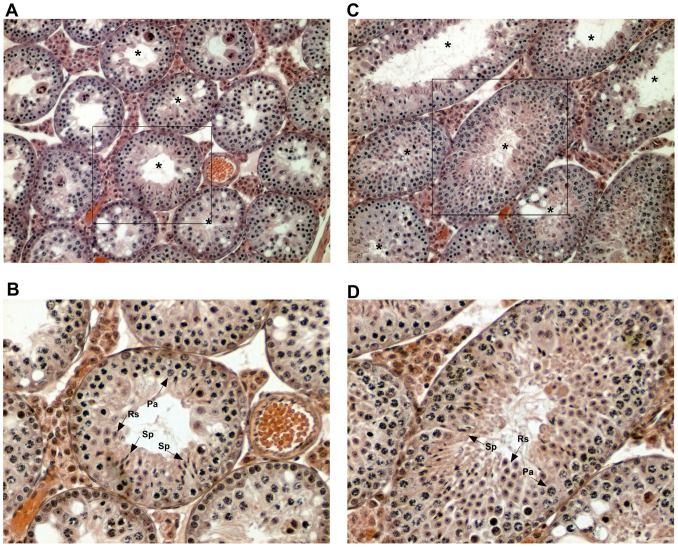
Testicular cross-section from (B6.PWD-Chr X.1s×PWK)F1. (**A**), (**B**) at nine (A, magnification 100x, B, 200x); (**C**), (**D**) at 25 weeks of age (C 100x, D 200x). Tubules in both the younger and older adult contained sperm (marked by asterisks in A, C and by “Sp” arrows in B, D). Pa, pachytene spermatocytes; Rs, round spermatids.

Hybrids resulting from the cross of a PWD female with a B6 male and of a PWD male with the B6.PWD-Chr X.1s subconsomic female are azoospermic [9], [10]. To determine how this differential segment of Chr X^PWD^ carrying the *Hstx2^PWD^* locus can influence hybrid fecundity in (PWK×B6)F1 genetic background regarding delayed fertility, the subconsomic female was crossed to PWK males and the resulting hybrids phenotyped at two time points. At the age of nine weeks, (B6.PWD-Chr X.1s×PWK)F1 male hybrids were azoospermic similarly as (B6.PWD-Chr X.1s×PWD)F1 and (PWD×B6)F1 hybrids (Table 5). However, testicular histology of these 9-week-old (B6.PWD-Chr X.1s×PWK)F1 males revealed immature sperm in some seminiferous tubules (Figure 3) and 25-week-old hybrids displayed a low number of spermatozoa in epididymides (Table 5), indicating a fertility delay. The later onset of fertility was again caused by meiotic arrest, as confirmed by quantitative analysis of the composition of primary spermatocytes of the 9- versus 25-week-old (B6.PWD-Chr X.1s×PWK)F1 males (Table 2; p = 0.045). In contrast, aged (B6.PWD-Chr X.1s×PWD)F1 males remained azoospermic (Table 5) due to arrested meiosis (Table 2). The delay of sperm production in (B6.PWD-Chr X.1s×PWK)F1 males further points to the role of PWK alleles outside of the *Hstx2* region.

**Table 5:** Comparison of fertility phenotypes of young and aged adult F1 hybrids.

	9-week-old			25-week-old		
Cross (female first)	TW	SCe	n	TW	SCe	n
PWD×B6	63	0.0	52	59*	0.0*	10
PWK×B6	110	1.7	12	104	3.0	4
B6.PWD-Chr X.1s×PWK	80	0.0	15	110	0.6	15

*p<0.05 compared to (B6.PWD-Chr X.1s×PWK)F1.

### Age-dependent Fertility of Adult STUP Hybrids

**Figure 4:**
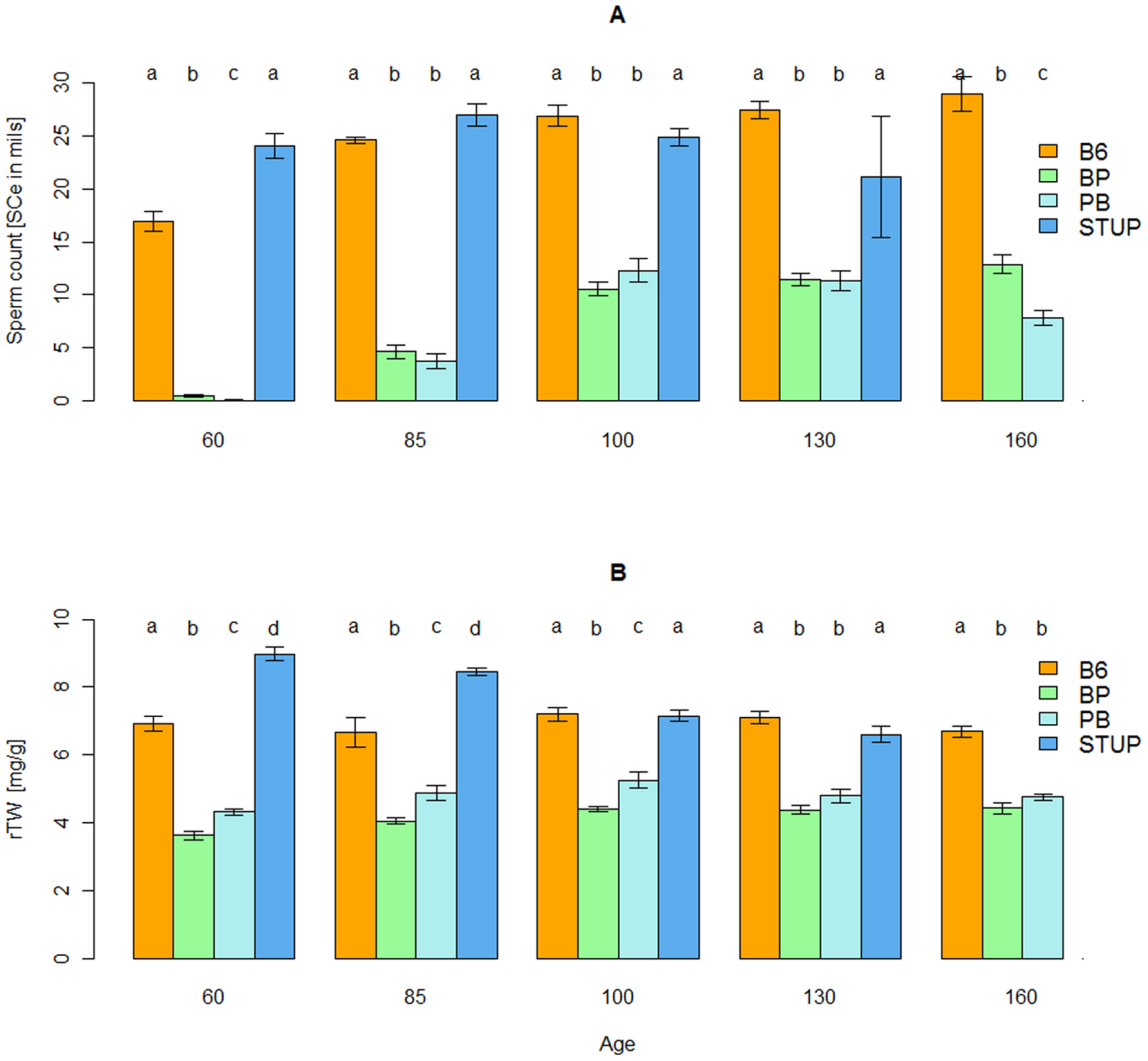
Age dependency of fertility parameters of adult STUP hybrids and the parental strains. (STUP×B6)F1 (PB, sample size n = 51), (B6×STUP)F1 (BP, n = 72), STUP (n = 38), and B6 (n = 55). (**A**), epididymal sperm count (SCe) versus age (days); (**B**), relative testis weight (rTW, mg TW per g BW) versus age (days). The letters symbolize values for the time point of the four genotypes named in ABC order from the left (value “a” for B6); the same letter used for the value of another genotype (e.g., “aa”) means that there is no significant difference between the two values (P>0.05, ANOVA) and a different letter used for another genotype (e.g., “b” for STUP) indicates a significant difference (P<0.05, ANOVA) between the values at these time points; log(SCe +1) was used for statistical analysis instead of SCe. SCe was significantly lower in young adult (60 to 85 days of age) versus aged adult F1 hybrids (100 to 130 days of age; see Table 6 for statistics), but even the aged hybrids did not reach the parameters of parental controls.

A possible delay of spermatogenesis has been detected in intersubspecific mouse F1 hybrids involving the wild Mmm-derived strain STUP [39]. The STUP strain has been developed from Mmm mice found about 150 kilometers from the site of origin of PWK. To verify the later onset, fertility parameters were measured in both types of STUP hybrids and parental controls at five time points spanning the interval between 60 and 160 days of age (Figure 4). The 60-days-old (STUP×B6)F1 and (B6×STUP)F1 hybrids carried no or a very few spermatozoa. Although SCe increased in hybrids and B6 with age, it reached a plateau by 85 days of age in the B6 males, but by 100 days of age in the hybrids (Figure 4A and Table 6). In addition, none of the two types of hybrids ever reached the SCe or rTW levels seen in the parental controls (Figure 4). These results suggest that delayed fertility is a fairly common phenotype of intersubspecific F1 mouse hybrids.

**Table 6:** Statistical evaluation of age dependency of fertility parameters in F1 males of STUP and B6 and their parents.

Age comp.	B6	STUP*	(STUPxB6)F1	(B6xSTUP)F1
TW	a,ab,b,b,b	a,a,a,a	a,ab,bc,bc,c	a,b,c,c,c
rTW	a,a,a,a,a	b,b,a,a	a,ab,b,ab,ab	a,ab,b,b,b
log(SCe+1)	a,b,b,b,b	a,a,a,a	a,b,d,d,c	a,b,c,c,c

The values at 60, 85, 100, 130, and 160 days of age were named by letters in ABC order from the lowest value; different letters for two time points in the same cell (e.g., a,b) indicate a significant difference (P<0.05, ANOVA) between these two time points. *Only four time points for STUP (no data at 160 days of age). See Figure 4 for SCe and rTW values.

### Lack of “*Overdrive”* Effect in (PWK×B6)F1 Hybrids

Young F1 hybrid males resulting from a certain cross of two *Drosophila pseudoobscura* subspecies are sterile due to incompatibility(ies) of a gene named *Overdrive* located on *D*. *pseudoobscura* Chr X, but some of these males become fertile and produce almost exclusively XX female offspring [5]. To evaluate whether the sterility-ensuring alleles undergo a drive in the aged mouse F1 hybrids, the offspring of (PWK×B6)F1 males were tested for transmission ratio distortions. The (PWK×B6)F1 males produced female and male pups in an approximately equal ratio, as 13 of 25 offspring were males. The segregation of the *Prdm9* alleles of proximal Chr 17 from (PWK×B6)F1 males was also similar, because 12 of 24 offspring tested inherited the PWK and 12 the B6 allele. Therefore, there is no evidence that the later onset of fertility in the (PWK×B6)F1 males is accompanied by preferential transmission of chromosomes carrying the major hybrid sterility loci from older males.

## Discussion

Although the *Prdm9* gene has been shown to participate in the complete sterility of (PWD×B6)F1, (STUS×B6)F1, and (B6×STUS)F1, as well as in the semifertility of (B6×PWD)F1 and (PWD×B6-*Prdm9^C3H^*)F1 [4], [24], its impact on semisterile hybrids carrying Mmm Chr X has remained elusive, despite their great importance for mouse speciation research [29], [35], [36]. We found a partial age-dependent meiotic arrest in semisterile (PWK×B6)F1 hybrids that was improved by the genetic manipulation of *Prdm9*, as well as Chr X. Thus the DMI(s) of *Prdm9^B6^* and a locus (or more likely loci) from Chr X^PWK^ also play a role in the fertility phenotypes of the semisterile (PWK×B6)F1 intersubspecific hybrids.

Our study demonstrated the importance of intersubspecific heterozygosity of proximal Chr 17 for the semisterility of (PWK×B6)F1 males; however, this region was not the cause of the phenotypic difference between (PWK×B6)F1 and (PWD×B6)F1 males. The *Hstx2* region had a detectable impact on the reproductive fitness of (PWK×B6)F1 hybrids, and it probably also contains polymorphism(s) between the PWK and PWD Mmm strains modifying the outcome of F1 fertility. Other loci probably affect the difference between fertility of (PWD×B6)F1 and (PWK×B6)F1; one of them could map distal to *Hstx2* on Chr X. Mmd displays polymorphisms in *Prdm9* affecting hybrid sterility; the B6 allele ensures complete sterility of (PWD×B6)F1 males, whereas the C3H and some other alleles do not [7], [11], [24]. Thus the DMI of *Prdm9* and the *Hstx2* region plays the main role in mouse F1 hybrid sterility, whereas their polymorphisms and other loci can mask or magnify the outcome of their interaction, confirming previous findings of incipient speciation between Mmm and Mmd [11], [29], [45], [46]. Our study shows that Mmm is polymorphic in alleles that are incompatible with *Prdm9* and/or *Hstx2*.

Chr X from all Mmm strains thus far investigated in sufficient detail (PWD, PWK, STUP, STUS) contribute to reduced sterility of intersubspecific hybrids. The genetic data from the mouse hybrid zone suggest the presence of multiple speciation gene(s) on Chr X [25], [26], [47].

The asynapsis of heterosubspecific chromosomes has been suggested to be the primary cause of hybrid sterility in the (PWD×B6)F1 and (PWD×SCHEST)F1 males [9], [10]. Asynapses were also detected in the (PWK×B6)F1 hybrids and their number was suppressed by removing *Prdm9^B6^*; asynapsis thus participates in multiple mouse hybrid infertility phenotypes.

The (PWK×B6)F1 hybrids are not just oligospermic; they also display a narrow window of age in which they can give rise to offspring. This age is delimited by delayed fertility (this report) and earlier cessation of fertility (Aylor DL, Bell TA, Detwiler D, Calaway ME, Crowley J, Pan W, Odet F, McMillan L, O’Brien DA, and Pardo-Manuel de Villena F, personal communication) to only about 3.5 months (approximately 3.5 to 7 months of age). Delayed fertility was also found in males carrying *Hstx2^PWD^* on (PWK×B6)F1 background, suggesting that the difference(s) modifying the (PWD×B6)F1 azoospermia map(s) out of the *Hstx2* region. Since a delayed onset of fertility was also demonstrated in (STUP×B6)F1 and (B6×STUP)F1 mouse hybrids (Figure 4), it is not an exceptional phenotype. Moreover, the sperm count of (STUP×B6)F1 males decreased after five months of age, supporting the view that the premature cessation of fertility is also a common phenomenon in intersubspecific hybrids.

The F1 male intersubspecific hybrids of the STUP strain did not display a reciprocal fertility bias as it is the case for PWK and PWD. However, STUP is not an exception, as there are other examples of strains lacking fertility differences in reciprocal intersubspecific crosses, including strains derived from the same locality as STUP, named STUS and STUF [39], or the PWB strain derived from a site near the origin of PWK and PWD [37]. The STUS and PWB strains produced fully sterile F1 hybrid males with B6 mice regardless of the direction of the cross, while the male offspring resulting from either reciprocal cross of STUF with B6 were fertile [37], [39], [46]. Thus, the reciprocal fertility bias probably exists independently of the fertility degree or delay.

Our findings should affect the design of crosses involving male hybrids of PWK and other wild-derived strains. The age-limited fertility phenotypes can be fully revealed only in the laboratory, not in males collected from the wild, where the age may be advanced or uncertain. Our data should therefore be taken into consideration when designing the studies and interpreting the results of fertility analyses of natural hybrid zones [35], [36]. As the PWK strain is the parental strain of the Collaborative Cross [48], the CC strains could be used to dissect the age-restricted fertility.

The hypothesis associating distorted segregation and unisexual reduction of hybrid fitness explains incompatibilities of two genomes as the outcome of the diversification of sex ratio distorters [49], [50]. Indeed, a single gene can participate in both transmission ratio distortion and reduced hybrid fertility. A DMI of the *Overdrive* gene causes hybrid sterility in (*Drosophila pseudoobscura* Bogota×*Drosophila pseudoobscura* USA)F1 males; old hybrids can regain fertility but transmit to offspring preferentially the *Overdrive*-carrying Chr X [5]. However, no skewed segregation of two major sterility alleles was found in the aged mouse (PWK×B6)F1 males. Admittedly, other chromosomes or hybrids may display distorted transmission, and the clarification of the relationship between mouse hybrid fertility and distorted segregation therefore requires additional work.

## Conclusions

Our results have several implications. First, the reduced fertility phenotype caused by the *Prdm9*–*Hstx2* incompatibility is restricted neither to full sterility nor to the genetic background on which it was discovered, but can be modified in both *cis* and *trans*. Second, asynapsis of heterosubspecific chromosomes during meiosis is dependent on *Prdm9* DMI and plays a role in multiple phenotypes reducing reproductive fitness. Third, male age must be considered to properly describe the phenotypes in all studies of hybrid fertility. Fourth, hybrid sterility alleles of the *Prdm9* and *Hstx2* genes do not undergo transmission ratio distortion in (PWK×B6)F1 males.

## Supporting Information

Text S1 **The ARRIVE (Animals in Research: Reporting** ***In Vivo*** **Experiments) guidelines checklist and details.**
(DOC)
