# Supplementary material for: Prdm9 Incompatibility Controls Oligospermia and Delayed Fertility but No Selfish Transmission in Mouse Intersubspecific Hybrids
Source: PLoS One. 2014 Apr 22;9(4):e95806. doi: 10.1371/journal.pone.0095806 (PMC3995920; doi:10.1371/journal.pone.0095806)
Supplement: Text S1 — The ARRIVE (Animals in Research: Reporting In Vivo Experiments) guidelines checklist and details. (DOC) [file pone.0095806.s001.doc]

**Petr Flachs^1^, Tanmoy Bhattacharyya^1^, Ondřej Mihola^1^, Jaroslav Piálek^2^,**

**Jiří Forejt^1^, Zdenek Trachtulec^1^**

**^1^**Department of Mouse Molecular Genetics, Institute of Molecular Genetics of the Academy

of Sciences of the Czech Republic, v.v.i., Prague, Czech Republic, **^2^**Research Facility Studenec, Institute of Vertebrate Biology, Academy of Sciences CR, Brno, Czech Republic.

**The ARRIVE guidelines checklist** (according to Kilkenny et al 2010).

**ITEM / no**

**Title / 1**

**Provide as accurate and concise a description of the content of the article as possible.**

*Prdm9* Incompatibility Controls Oligospermia and Delayed Fertility but no Selfish Transmission in Mouse Intersubspecific Hybrids

**Abstract / 2**

**Provide an accurate summary of the background, research objectives, including details of the species or strain of animal used, key methods, principal findings and conclusions of the study.**

-Section ABSTRACT: background, research objectives, including the species and strains used, principal findings and conclusions of the study.

Key methods: F1 hybrid males resulting from the crosses of mouse strains derived from two mouse subspecies (including *Prdm9* null and transgenic) and control mice (littermates and parental strains) underwent fertility measurements, including the number of offspring per female per month and (after dissection) testes and body weight, sperm count, and testicular immunocytochemistry.

**INTRODUCTION**

**Background / 3**

**a. Include sufficient scientific background (including relevant references to previous work) to understand the motivation and context for the study, and explain the experimental approach and rationale.**

-Paragraphs 1 to 4

**b. Explain how and why the animal species and model being used can address the scientific objectives and, where appropriate, the study’s relevance to human biology.**

-Paragraph 5

**Objectives / 4**

**Clearly describe the primary and any secondary objectives of the study, or specific hypotheses being tested.**

-Paragraph 4 to 5

**METHODS**

**Ethical statement / 5**

**Indicate the nature of the ethical review permissions, relevant licences (e.g. Animal [Scientific Procedures] Act 1986), and national or institutional guidelines for the care and use of animals, that cover the research.**

- Paragraph 1 (Ethics statement)

**Study design / 6**

**For each experiment, give brief details of the study design including:**

**a. The number of experimental and control groups.**

Indicated in the Tables.

**b. Any steps taken to minimise the effects of subjective bias when allocating animals to treatment (e.g. randomisation procedure) and when assessing results (e.g. if done, describe who was blinded and when).**

Mice were taken for analysis by cage, not by genotype. About 15 % of mice were dissected by a “blinded” researcher because the mice were (re)genotyped after phenotyping (tag loss, time scheduling). Most of the time, the researcher performing the experiments had no knowledge of the previous results. In addition, delayed puberty was detected and analyzed independently in two different laboratories.

**c. The experimental unit (e.g. a single animal, group or cage of animals).**

Four males for each immunocytochemistry experiment (mostly two pairs of littermates with a different genotype); a cage of males for all the others.

**d. A time-line diagram or flow chart can be useful to illustrate how complex study designs were carried out.**

Not complex; one generation crosses (PWK with B6 congenic or (sub)consomic strain) indicated in each Table and one N2 (BC1) cross.

**Experimental procedures / 7**

**For each experiment and each experimental group, including controls, provide precise details of all procedures carried out. For example:**

**a. How (e.g. drug formulation and dose, site and route of administration, anaesthesia and analgesia used [including monitoring], surgical procedure, method of euthanasia). Provide details of any specialist equipment used, including supplier(s).**

**b. When (e.g. time of day).**

**c. Where (e.g. home cage, laboratory, water maze).**

**d. Why (e.g. rationale for choice of specific anaesthetic, route of administration, drug dose used).**

Mice were sacrificed in the morning by cervical translocation in the laboratory; all mice used for testicular immunocytochemistry were first anesthetized by CHCl_3_ vapor. Surgery was not performed on live animals.

**Experimental animals / 8**

**a. Provide details of the animals used, including species, strain, sex, developmental stage (e.g. mean or median age plus age range) and weight (e.g. mean or median weight plus weight range).**

-Paragraph 2 (Mice and genotyping). The mean values and the number of mice for each genotype are indicated in the Tables.

**b. Provide further relevant information such as the source of animals, international strain nomenclature, genetic modification status (e.g. knock-out or transgenic), genotype, health/immune status, drug or test naive, previous procedures, etc.**

-Paragraph 2. Healthy animals were drug- and test- naive; most genotyping was performed on tail clips before phenotyping.

**Housing and husbandry / 9**

**Provide details of:**

**a. Housing (type of facility e.g. specific pathogen free [SPF]; type of cage or housing; bedding material; number of cage companions; tank shape and material etc. for fish).**

The experiments were conducted in the SPF facility of IMG in Prague except for the age dependency of hybrids of STUP and B6 (these mice, including parental controls, were bred and phenotyped in the barrier-free facility in Studenec).

**b. Husbandry conditions (e.g. breeding programme, light/dark cycle, temperature, quality of water etc for fish, type of food, access to food and water, environmental enrichment).**

**c. Welfare-related assessments and interventions that were carried out prior to, during, or after the experiment.**

All mice were allowed free access to food and acidified water. In Prague, mice were fed by ROD 18 (LASvendi, Soest, Germany) and kept in open cages on aspen bedding PK3 (LASvendi) in a controlled temperature of 22°C, humidity of 55%, and light/dark cycle of 12/12 hours. Environmental enrichment (plastic hideouts) was provided in the breeding cages. F1 males were weaned at four weeks of age. In Studenec, mice were maintained on pelleted food ST1 (VELAZ, Prague, Czech Republic) at 20–22°C in Perspex cages with a 14/10-hour light/dark cycle.

**Sample size / 10**

**a. Specify the total number of animals used in each experiment, and the number of animals in each experimental group.**

Indicated in the Tables.

**b. Explain how the number of animals was arrived at. Provide details of any sample size calculation used.**

**c. Indicate the number of independent replications of each experiment, if relevant**

For phenotyping of testicular weight and sperm count: males from two litters were genotyped and phenotyped; in case the two genotypes differed in a fertility parameter, additional litters were produced. The size of the additional litter was proportional to the phenotypic difference. For immunocytochemistry, testicles from two to three males of each genotype were used, as the immunocytochemistry data were in agreement with the data on other fertility parameters (testis weight and sperm count were proportional to the strength of meiotic arrest). About 50 pachytene cells were counted for each male (biological replicas) from one or more slides (technical replicates).

**Allocating animals to experimental groups / 11**

**a. Give full details of how animals were allocated to experimental groups, including randomisation or matching if done.**

**b. Describe the order in which the animals in the different experimental groups were treated and assessed.**

Random order of cages and animals for phenotyping; littermate controls.

**Experimental outcomes / 12**

**Clearly define the primary and secondary experimental outcomes assessed (e.g. cell death, molecular markers, behavioural changes).**

- Paragraph 3 (Phenotyping and statistics): primary: animal Id; cage Id; date of birth, date of sacrifice, genotype, BW, TW, dilutions, cell counts, offspring number and date of birth; 2ndary: age, SC, rTW, % of cell populations, offspring per female per month.

**Statistical methods / 13**

**a. Provide details of the statistical methods used for each analysis.**

- Paragraph 3 (Phenotyping and statistics)

**b. Specify the unit of analysis for each dataset (e.g. single animal, group of animals, single neuron).**

A group of males with the same genotype and age served as experimental unit in each test.

**c. Describe any methods used to assess whether the data met the assumptions of the statistical approach.**

Not tested.

**RESULTS**

**Baseline data / 14**

The health status of the animals at IMG was monitored throughout the experiments by a health surveillance program according to Federation of European Laboratory Animal Science Associations (FELASA) guidelines. The SPF mice were free of all viral, bacterial, and parasitic pathogens listed in the FELASA recommendations, except for *Stenotrophomonas maltophilia* (one positive mouse of 140 tested).

**Numbers analysed / 15**

**a. Report the number of animals in each group included in each analysis. Report absolute numbers (e.g. 10/20, not 50%2).**

Indicated in the Tables.

**b. If any animals or data were not included in the analysis, explain why.**

All animals included.

**Outcomes and estimation / 16**

**Report the results for each analysis carried out, with a measure of precision (e.g. standard error or confidence interval).**

Indicated in the Tables and Figures, statistical outputs (p-values) in the text, Tables or Figure legends.

**Adverse events / 17**

**a. Give details of all important adverse events in each experimental group.**

Some hybrid males (regardless of phenotype) were aggressive.

**b. Describe any modifications to the experimental protocols made to reduce adverse events.**

The aggressive males were housed in separate cages.

**DISCUSSION**

**Interpretation/ scientific implications / 18**

**a. Interpret the results, taking into account the study objectives and hypotheses, current theory and other relevant studies in the literature.**

- paragraphs 1 to 3

**b. Comment on the study limitations including any potential sources of bias, any limitations of the animal model, and the imprecision associated with the results.**

No potential sources of bias or imprecision are known. The possible limitation of intersubspecific hybrids as an animal model is the translatability to non-hybrid research.

**c. Describe any implications of your experimental methods or findings for the replacement, refinement or reduction (the 3Rs) of the use of animals in research.**

The number of animals used for experiments was reduced by taking multiple phenotypic measurements on each male and regenotyping instead of discarding in the case of tag loss.

**Generalisability/ translation / 19**

**Comment on whether, and how, the findings of this study are likely to translate to other species or systems, including any relevance to human biology.**

- paragraph 4: relevance to other studies

**Funding / 20**

**List all funding sources (including grant number) and the role of the funder(s) in the study.**

- section Funding

**- References :**

Kilkenny C, Browne WJ, Cuthill IC, Emerson M, Altman DG (2010) Improving Bioscience Research Reporting: The ARRIVE Guidelines for Reporting Animal Research. PLoS Biol 8(6): e1000412. doi:10.1371/journal.pbio.1000412
